# Adaptive network steganography using deep learning and multimedia video analysis for enhanced security and fidelity

**DOI:** 10.1371/journal.pone.0318795

**Published:** 2025-06-05

**Authors:** Chunhong Han, Tao Xue

**Affiliations:** Department of Information Engineering, Jiaozuo Normal College, Jiaozuo, China; UNISA: Universita degli Studi di Salerno, ITALY

## Abstract

This study presents an advanced adaptive network steganography paradigm that integrates deep learning methodologies with multimedia video analysis to enhance the universality and security of network steganography practices. The proposed approach utilizes a deep convolutional generative adversarial network-based architecture capable of fine-tuning steganographic parameters in response to the dynamic foreground, stable background, and spatio-temporal complexities of multimedia videos. Empirical evaluations using the MPII and UCF101 video repositories demonstrate that the proposed algorithm outperforms existing methods in terms of steganographic success and resilience. The framework achieves a 95% steganographic success rate and a peak signal-to-noise ratio (PSNR) of 48.3 dB, showing significant improvements in security and steganographic fidelity compared to contemporary techniques. These quantitative results underscore the potential of the approach for practical applications in secure multimedia communication, marking a step forward in the field of network steganography.

## 1. Introduction

In the modern digital age, characterized by the proliferation of the Internet and burgeoning digital technologies, the seamless exchange of information is foundational to contemporary societal constructs. Yet, as the facility and rapidity of information transmission burgeon, the conundrum of information security looms large. Given this backdrop, network steganography has been catapulted to prominence as a quintessential modality for clandestine communication [1–3]. As a mechanism adept at obfuscating information within a medium, it remains undetectable to all but the intended interlocutors, rendering it invaluable in the realms of network, information, and computer security.

The annals of network steganography are replete with a plethora of techniques, ranging from text and image to audio and video steganography [4,5]. While text steganography embeds covert information within textual mediums through the insertion of indiscernible markers [6], image steganography hinges on embedding covert messages within images, leveraging methods such as LSB steganography and DCT steganography [7]. Audio steganography, conversely, subtly manipulates imperceptible auditory attributes to house covert data [8], and video steganography modifies latent video parameters to conceal information [9].

Nevertheless, the dynamic nature of network landscapes coupled with advancements in multimedia technologies presents both opportunities and challenges for traditional steganographic techniques. Moreover, emerging multimedia technologies, while offering myriad embedding possibilities, also pose intricate challenges to the formulation and execution of steganographic algorithms. Common challenges include: (1) Invisibility: existing methods often result in visible distortion in cover images or videos, which allows steganography analysis tools to detect the presence of hidden data. (2) Robustness: Many traditional steganography algorithms fail when the carrier media is subjected to common signal distortions, such as compression (e.g., JPEG or H.264), resizing, or adding noise. This can leave hidden data vulnerable to detection or corruption. (3) Limited embedding capacity: previous approaches often struggle to achieve high embedding capacity while maintaining the quality of the cover media, thus limiting the amount of information that can be effectively hidden.

In response to these intricacies, recent innovations have beckoned steganographic methodologies anchored in deep learning, artificial intelligence, and quantum cryptography. Adeeb and Kabudian [10] explored new frontiers in steganography, focusing on Arabic text concealment using deep learning. Their study employed Natural Language Processing algorithms and Artificial Intelligence theories to hide secret data within Arabic poetry, showcasing a 45% increase in storage capacity. Li et al. [11] enhanced the detection of generative linguistic steganography through their ELM method, addressing explicit and latent text word relations to improve performance. Tudorache et al. [12] presented a quantum steganography approach based on the B92 quantum protocol, utilizing grayscale images and quantum circuits for secure data transmission. Gadicha et al. [13] emphasized a multimode approach to data encryption in images through quantum steganography, contributing to the broader landscape of information security. In the comprehensive resolution of network security issues, we recognize the importance of exploring diverse solutions, including those beyond traditional algorithmic methods. The landscape of network security is multifaceted, and hardware-based solutions play a pivotal role in fortifying digital ecosystems. Fragkos et al. [14] conducted in-depth research in the field of Artificially Intelligent Electronic Money, exploring the use of physical unclonable functions (PUF) for authentication and encryption in e-Cash transactions. The study introduces innovative artificial intelligence methods within the PUF-Cash scheme, showcasing the selection of trusted third parties (TTP) and the distribution of withdrawal amounts to optimize performance and privacy. Each of these avant-garde techniques, from deep learning-based to quantum cryptography-based steganography, has its own set of merits and demerits, necessitating nuanced research and refinement [15–18].

To address these challenges, the proposed adaptive network steganography model combines deep learning with multimedia video analytics to address these shortcomings. The proliferation of digital technologies and the internet has led to an exorbitant growth of multimedia information, as highlighted by Kar et al [19]. Our research addresses these challenges by proposing an adaptive network steganography paradigm that integrates deep learning methodologies with multimedia video analysis.Our model enhances invisibility by using advanced Generative Adversarial Networks (GANs), which generate visually indistinguishable steganographic images/videos, ensuring that the embedded data is imperceptible to the human eye and robust to detection algorithms. We improve the robustness by introducing advanced techniques such as gradient penalty in the WGAN-GP framework, which makes the hidden data more resistant to compression, noise, and other signal distortions and ensures the integrity of the embedded information even in challenging environments. The proposed approach also achieves higher embedding capacity, allowing for larger amounts of hidden information while maintaining high-quality video and image output. By directly addressing these critical limitations, our proposed approach is more suitable for practical real-world applications such as video streaming and secure communication in digital media platforms.

The purpose of this study is to develop an adaptive network steganography framework that utilizes deep learning and multimedia video analysis to enhance the security and generality of steganography techniques. Its significance lies in its ability to dynamically adjust to various video formats, improving robustness to common signal distortions and detection algorithms. This study is crucial for secure communication in the digital age, providing a powerful solution for the dissemination of secret information in multimedia content.

## 2. State of the art

### 2.1. Generating adversarial networks

**Fig 1:**
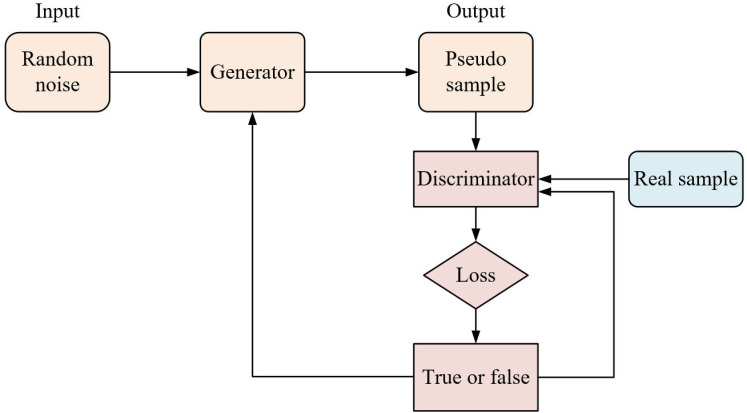
Structure of GAN.

Generative adversarial network (GAN) is a neural network model based on the idea of zero-sum game [20]. Its structure is shown in Fig 1. The term “generator” denotes a key component of the GAN architecture. The generator is responsible for generating synthetic data, such as images or videos, by transforming random noise or latent vectors into realistic-looking samples.

The GAN samples random noise from a distribution and feeds it to the generator [21], which outputs a “pseudo” sample that is passed to the discriminator along with the “true” sample to give a discriminant result. Ideally, the generator learns the real data distribution completely, so that the discriminator cannot accurately identify the data source [22]. Therefore, the optimization objective function in the training process of GAN is


$$\min_{A}\;\max_{D}\;V(D,A)=E_{i\sim u_{\text{data }}(i)}\left[logD(i)\right]+E_{k\sim u_{\text{noise }}(i)}\left[log\left(1-D\left(A(k)\right)\right)\right],$$ (1)

Where, D(i) is the probability of discriminating *i* as a true sample. A(k) is the generated sample generated from the input noise *k*. The notation $u_{\text{data }}(i)$ was intended to represent the empirical data distribution from which real data samples are drawn. This notation implies that *i* is a sample drawn from the distribution $u_{\text{data}}$, which does not functionally depend on *i* itself but rather denotes that *i* is sampled according to the distribution $u_{\text{data }}$.

To achieve this optimization, GANs rely on the generation of random noise, which is a critical component of the process. The random noise, or latent vectors, is sampled from a Gaussian distribution with a mean of 0 and a standard deviation of 1. This noise is then fed into the generator network, which transforms these vectors into synthetic data samples that aim to mimic the distribution of real data. The creation of this random noise involves random sampling from the Gaussian distribution, forming these samples into vectors, and then using these vectors as input to the generator. This process is essential for the diversity and realism of the generated samples, and it plays a pivotal role in the training and effectiveness of the GAN model.

The deep convolutional generative adversarial network refers to the principle of the original GAN, and the convolutional neural network is the main body of its structure. The full convolutional structure is used in the network model instead of the fully connected and pooling layers, and the use of batch standard normalization layers solves the training problem of the model, so that the quality of generated samples and the convergence speed are greatly improved.

WGAN (Wasserstein GAN), as one of the derivative models of the original GAN, replaces the Jensen-Shannon divergence with Wasserstein distance and improves the loss function to solve the problem of gradient disappearance and instability in the training process of the original generative adversarial network, while ensuring the diversity of the generated data samples. The method of Wasserstein distance metric is shown in Eq (2)


$$M\left(U_{r},U_{a}\right)=\inf_{\Pi \left(U_{r},U_{a}\right)}\;E(i,j)\sim \gamma [∥i-j∥],$$ (2)

Where, $\Pi \left(U_{r},U_{a}\right)$ denotes the set of all joint distributions of the true data distribution $U_{r}$ and the generated data distribution $U_{a}$. γ[∥i−j∥] denotes the true sample *i* and the generated sample *j* obtained by sampling from γ. The two distances ∥i−j∥ are calculated according to Eq (2), and the expectation E(i,j)~γ[∥i−j∥] of the *i* and *j* distances under γ is obtained. Taking a lower bound on this expectation yields $M\left(U_{r},U_{a}\right)$. After further mathematical calculations, the Wasserstein distance approximation formula is obtained as


$$M\left(U_{r},U_{a}\right)=\frac{1}{Z}{∥}_{f_{\omega }}\underset{L}{wgi}\;\leq Z=E_{i}\sim U_{r}\left[f_{\omega }(i)\right]-E_{i}\sim U_{a}\left[f_{\omega }(i)\right],$$ (3)

Where *Z* denotes the first-order Lipchitz constant of the function *f*. The change in Z causes a *Z*-fold change in the gradient of the network, but does not affect the direction of the gradient, and WGAN trains the discriminator D and the generator *A* as in Eq (4)


$$\min_{A}\;\max_{D}\;\;L_{WGAN}(D,A)=\underset{i\sim \sim {\mathbb{P}}_{a}}{E}\left[D\left(\tilde{i}\right)\right]-\underset{i\sim {\mathbb{P}}_{r}}{E}\left[D(i)\right].$$ (4)

Due to the interaction between the weight constraint and the loss function, the WGAN model suffers from the difficulties of network training, gradient explosion and the gap between the quality of the generated image samples and the real image quality. The improved model WGAN-GP replaces the weight clipping method with gradient penalty, which essentially adds a penalty term to the original loss function to correlate the gradient with K in order to preserve the 1-Lipschitz. The WGAN-GP training discriminator D and generator *A* are given in Eq (5)


$$\min_{A}\;\max_{D}\;\;L_{WGAN-GP}(D,A)=\underset{i\sim U_{a}}{E}[D(\tilde{i})]-\underset{i\sim U_{r}}{E}[D(i)]+\lambda_{\hat{i}\sim U_{\hat{i}}}^{E}\left[{\left(∥\hat{i}D(\hat{i}){∥}_{2}-1\right)}^{2}\right].$$ (5)

The last term of the gradient penalty term for network regularization is added to the WGAN training model. For the gradient penalty term $\lambda_{\hat{i}\sim U_{\hat{i}}}^{E}\left[{\left(∥\hat{i}D(\hat{i}){∥}_{2}-1\right)}^{2}\right]$ denotes the gradient penalty coefficient. $\hat{i}$ is a random interpolation sample between the real data sample $U_{r}$ and the generator’s generated data sample $U_{a}$: $\hat{i}=\varepsilon i_{r}+(1-\mathrm{\backslash varepsilonwidetilde}i_{a}$. Where ε represents the Uniform [0, 1] random number. $i_{r}$~ $U_{r}$ represents the sample from the real data. $\tilde{i_{a}}$~ $U_{a}$ represent the generated data samples from the generator.

The WGAN-GP model applies the gradient penalty strategy to the training network. This improves the network convergence performance and the quality of the generated image samples, making the network training more stable and more generalizable under different network architectures. Therefore, the model in this paper uses WGAN-GP to solve the difficulties of network training not easily converged, gradient explosion and poor quality of generated image samples that exist in existing image steganography models based on generative adversarial networks in the image steganography process.

### 2.2. Semi-generative steganography

**Fig 2:**
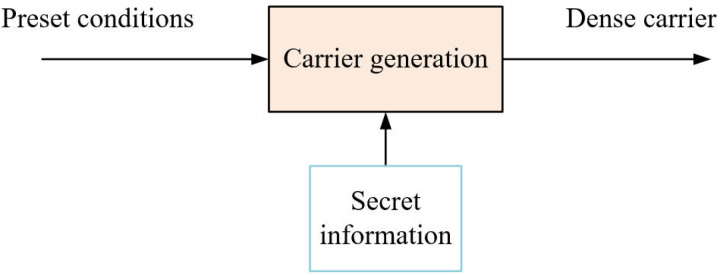
Semi-generative steganography.

Generative information concealment refers to the methodology wherein, in the absence of a pre-designated original carrier, a confidential carrier is formulated directly from the confidential information based on established protocols. It is imperative to note that this confidential carrier might not depict the actual empirical world. However, it ought to be indistinguishable when juxtaposed with standard content. Typically, generative methodologies refrain from pre-determining the original carrier. In contrast, semi-generative message concealment approaches delineate pre-established parameters for carrier formulation. Subsequent to this, they produce the confidential carriers predicated on the confidential message, adhering to the pertinent generative protocols. It is noteworthy that the resultant cryptographic carriers pertain to a distinct category, as delineated in Fig 2.

Contrary to the carrier-modified steganographic paradigm, the semi-generative steganographic methodology for video is primarily motivated by the need to handle confidential data. This approach employs a generator to directly produce video exemplars embedded with secret data, thereby circumventing the alteration of the original carrier. It is tailored for a distinct category of video carriers and is underpinned by its singular theoretical framework and technique for information concealment. Additionally, it assimilates certain outcomes from contemporary carrier-modified steganographic paradigms. The semi-generative steganographic framework for video can be efficaciously employed in social software, streaming media platforms, and digital storage utilities, leveraging the video medium as a subterfuge for clandestine data, thereby facilitating covert communication or storage.

## 3. Methodology

The proposed method centers on an adaptive steganographic framework that leverages deep convolutional generative adversarial networks (GANs) to dynamically adjust steganographic parameters in response to the dynamic foreground, stable background, and spatio-temporal complexities present in multimedia videos. The core of the proposed method lies in an adaptive steganographic framework based on deep convolutional Generative Adversarial Networks (GANs). This framework is capable of fine-tuning steganographic parameters in response to the dynamic foreground, stable background, and spatio-temporal complexities of multimedia videos. This dynamic adjustment capability enables our model to adapt to different video formats, as it can be optimized for the unique content and structural characteristics of each format. Secondly, the method proposed in this paper utilizes spatio-temporal masking to distinguish between the dynamic foreground and static background within video frames. This distinction allows the model to recognize and adapt to changes in dynamic and static elements across different types of video formats, thereby embedding secret information while maintaining video quality.

The adaptive network steganography based on the combination of deep learning and multimedia video analysis algorithms proposed in this paper consists of three major parts: Video generation, digitized Cardan generation and information embedding. The video generation needs to include the participation of the generator network and two discriminator networks, and the digitized Cardan generation and information embedding are based on the generation of the mask and foreground.


g=w⊙f+(1−w)⊙h (6)

Where, g is the sample. w is the spatio-temporal mask. f and h are the foreground information and the background information, respectively. ⊙  is the Hadamard product [23].

**Fig 3:**
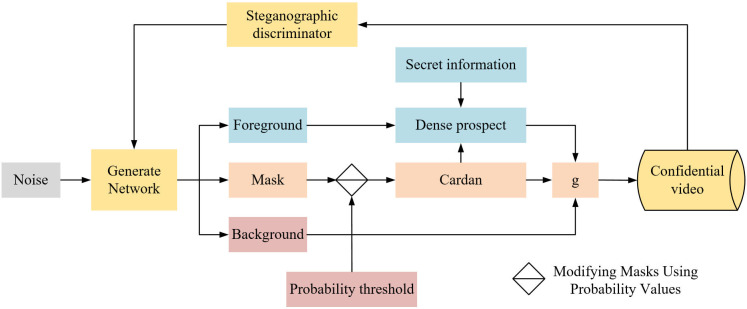
The process of generating video with encryption.

The process of generating a video containing a secret message in this paper is shown in Fig 3.

As shown in Fig 3, the term “Confidential video” refers to the “Steganographic video,” which is the output video containing embedded secret messages. And “Dense prospect” refers to “dynamic foreground”. It corresponds to the dynamic content in the video frames, represents the pixel regions that undergo motion, and is the key area for embedding secret information. “g” introduced in Equation (6), represents the synthesized sample video frame generated using the Hadamard product to blend the modified foreground (*f*) and the background (*h*) with the spatio-temporal mask (*w*).

The process of message hiding conforms to the basic idea of traditional Cardan. The sender adaptively defines a mask called digital Cardan by setting a steganography threshold to determine the specific locations where the message is hidden in the foreground information. The “digital Cardan” is an innovative concept in our steganographic methodology, inspired by the traditional Cardan grille used in physical steganography. In the context of digital video steganography, our “digital Cardan” adapts this concept to the digital domain. It serves as a digital mask that determines which pixels or regions within a video frame will be modified to embed secret data. The secret message will be embedded directly into the lowest valid bits of the pixel points at these locations, enabling the conversion from traditional Cardan to digital Cardan. The Hadamard product is then used for sample g synthesis through Eq (2). The video containing the secret video will be transmitted to the receiver through the public channel, and the receiver extracts the secret information using the Cardan obtained from the secret channel in advance. This ensures both the security of the secret information and the logical rationality before and after the video synthesis.

### 3.1. Digital Cardan generation and information embedding

To enhance security, the proposed approach integrates dynamic parameter adjustments during training, adaptive mask modifications, and the Hadamard product. These measures intricately weave the steganographic payload into video frames, minimizing alterations to overall video statistics. A steganography discriminator is introduced to enhance security by distinguishing videos containing hidden information. The digital Cardan generation process involves specific thresholds and adaptive mask modification. The combined effect of these techniques ensures that the generated steganographic multimedia maintains visual and perceptual similarity to the original content, thereby enhancing its resistance against advanced detection methods. In addressing dynamic changes in video content, our method demonstrates a degree of adaptability. By introducing dynamic parameter adjustments and adaptive masks, we can flexibly adjust steganographic operations as the video undergoes changes. This adaptive design enables our algorithm to effectively handle dynamic variations in video content, maintaining high steganographic performance.

In the Cardan-based information hiding method, Cardan is used as the key for secret information embedding and extraction, but the traditional Cardan is relatively simple for secret information processing. A digital Cardan steganography scheme is proposed for the characteristics of dual-stream video generation model.

In this paper, the mask is modified by analyzing the digital characteristics of the information corresponding to the motion and position of each pixel in the mask and setting specific steganography thresholds to generate the digital Cardenas. Due to the sigmoid function used in the last layer of the generation network, the values corresponding to the pixel motion information corresponding to the mask are in the range of 0 to 1. The value of the motion information is proportional to the magnitude of the pixel motion in the corresponding foreground, and the mask is modified in terms of both embedding rationality and embedding capacity as follows:


$$\mathrm{\backslash [}{U_{x,y}^{t}}^{\prime }=\left\{ \begin{array}{c} 1,U_{x,y}^{t}≽\partial \\ U_{x,y}^{t},U_{x,y}^{t}<\partial \end{array}\right.\;\mathrm{\backslash ]}$$ (7)

where, ${U_{x,y}^{t}}^{\prime }$ is the modified motion probability value. $U_{x,y}^{t}\;$ represents the motion probability value of the nth tensor corresponding to the *x*-th row and *y*-th column in the first dimension of the mask. ∂ is the set probability threshold.

Since only the first channel of the video frame image is embedded in the embedding process, the processing of foreground and mask is also performed only in its last dimension. If the size of foreground and mask is 32 ×  64 ×  64 ×  3, the size of the part to be data processed for both is 32 ×  64 ×  64 ×  1. Each generated mask is generated based on random noise drive, and the embedding position is selected by setting the threshold ∂ , placing the one greater than the probability threshold at 1, adaptively generating the digitized Cardan, and then corresponding to the specific position of foreground for LSB embedding. In order to reduce the feature changes brought by modifying the foreground pixel information, the embedding is carried out by randomly adding or subtracting 1, so as to achieve the purpose of optimal embedding. The specific embedding method and process are shown as


$$\mathrm{\backslash [}{f_{x,y}^{t}}^{\prime }=\left\{ \begin{array}{c} f_{x,y}^{t}+e_{x,y}^{t},f_{x,y}^{t}\neq w_{x,y}^{t}\\ f_{x,y}^{t},f_{x,y}^{t}=w_{x,y}^{t} \end{array}\right.\;\mathrm{\backslash ]}$$ (8)

where ${f_{x,y}^{t}}^{\prime }$ is the foreground information after embedding. $f_{x,y}^{t}$ represents the information corresponding to the *x*-th row and *y*-th column under the first dimension in the foreground. $e_{x,y}^{t}$ represents the modification method of the information corresponding to the *x*-th row and *y*-th column under the first dimension in the modified part of the foreground. If the lowest bit of the corresponding position in the foreground is different from the embedding information, the embedding is performed according to the above-mentioned modification method.

When the value of the desired modification position in the foreground is 0 or 255, the modification skips the point and embeds it at the next position. For transmission, the message sender does not need to transmit the network training model to the receiver, but transmits only the generated digitized Cardan to the receiver through the secret channel. When the message receiver receives the steganography video from the public channel, the secret information is extracted from the video frames without loss by overwriting each frame of the video with the digitized Cardan.

To ensure the integrity of the original multimedia content, we employ an adaptive digital Cardan steganography scheme in the proposed method. In the generation and information embedding processes of digital Cardan, we intricately embed the steganographic payload into video frames by adjusting dynamic parameters and adaptively modifying masks. The design of this process aims to minimize modifications to the overall video statistical characteristics, ensuring that the generated steganographic multimedia maintains visual and perceptual similarity to the original content, thus preserving the integrity of the original multimedia content. It is important to note that the algorithm’s performance may be influenced by specific network characteristics such as bandwidth, latency, and stability, which will be further discussed in the context of real-world network applications.

### 3.2. Generator structure of adaptive network steganography

The generator network is initialized with a low-dimensional noise variable, represented as z, drawn from a prescribed distribution, such as the Gaussian distribution. The structural design of the generator network, as outlined in this study, is predicated on several key criteria:

(1) The objective is for the low-dimensional noise to be capable of generating videos imbued with high-dimensional data.
(2) Given that videos accentuate the dynamic attributes of objects, leading to observable displacements in contrast to static images, it is imperative for the generator to encapsulate the dynamic properties of said objects.
(3) The generator can directly encapsulate spatiotemporal relationships, avoiding the separation of temporal and spatial components.

**Fig 4:**
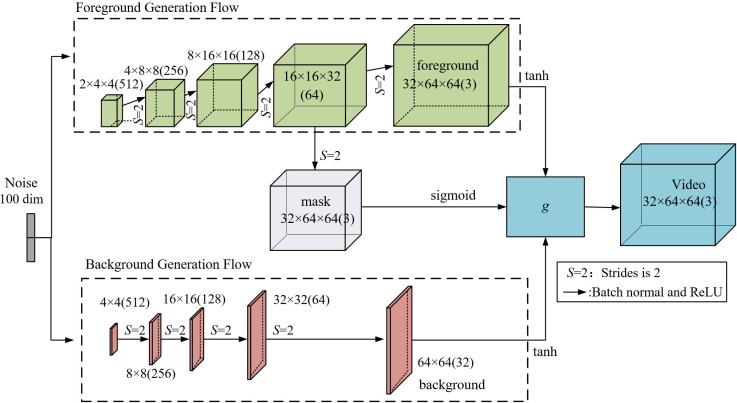
Generator network structure.

Upon critical evaluation of various extant video generation models rooted in neural network architectures, this study has elected to utilize a video dual-stream generation network. This network is predicated on an enhanced model equipped with a transposed convolutional network, characterized by a convolutional kernel dimension of 3 ×  3 ×  3 and a stride length of 2. The detailed architecture is illustrated in Fig 4, where the parenthetical numerals indicate the channel dimensions.

Within the context of video content, the ambient environment encompassing the objects is predominantly static, with primary motion typically attributed to the objects being captured. The dual-stream architectural approach, cognizant of this characteristic, strategically formulates the modus operandi and foundational tenets for video generation. For each pixel’s spatial positioning and temporal increment, a decision between foreground and background information is made via a temporal mask. The foreground encapsulates the video’s kinetic data, whereas the background denotes the static backdrop. To synthesize the background information through the video’s temporal series, the rear ground assimilates data from the static pixels within the video frame through bidimensional convolution, consequently producing a temporally consistent planar image.

Ensuring spatio-temporal consistency between the foreground and background is achieved through a series of operations that integrate the dynamic foreground with the temporally stable background. This integration is facilitated by a spatio-temporal mask, which serves as a pivotal tool in our adaptive network steganography. The mask, generated based on the motion probabilities of each pixel, allows for the selective embedding of secret data into the foreground while preserving the background’s integrity. By applying the mask, we can directly encapsulate spatio-temporal relationships without separating temporal and spatial components, thus maintaining the natural dynamics and spatial coherence of the video content. The generator network is designed to produce outputs that respect these spatio-temporal relationships. It does so by utilizing a dual-stream architecture, where one stream is responsible for generating the foreground data and the other for the background data. Each stream operates on different aspects of the video content, with the foreground stream capturing the kinetic data and the background stream synthesizing the static environment. The streams are then merged using the spatio-temporal mask to create a unified video frame that maintains the illusion of a single, continuous scene. To further enhance the spatio-temporal consistency, we employ a Hadamard product during the sample synthesis phase. This mathematical operation aligns the foreground and background information in a way that respects their respective contributions to the overall video frame. The result is a video sequence where each frame is a coherent blend of dynamic and static elements, preserving the natural flow of the video while allowing for the covert transmission of information.

The foreground is conceptualized as a quad-dimensional spatio-temporal tensor, epitomizing the spatio-temporal attributes of every pixel. The framework employed for foreground generation, as delineated in this study, is predicated on a singular-stream architecture. Conversely, the rear ground’s generative architecture mirrors that of conventional image generation networks. To demarcate the synthesis procedure of the spatio-temporal mask from the foreground, the concluding convolutional operation employs a convolutional layer with distinct parameters relative to the foreground. Notably, the mask retains weight congruence with the foreground across the residual convolutional layers.

In summary, we propose a novel approach to adaptive network steganography that integrates deep learning with multimedia video analysis. Our method involves the generation of both the foreground and background as distinct components within a video. Specifically,

Foreground and Background Generation: We employ a semi-generative approach, wherein a generator network creates video frames containing secret data without modifying an original carrier. The foreground and background are effectively separated using a spatio-temporal mask. The mask is employed to distinguish between dynamic (foreground) and static (background) elements, ensuring that the foreground contains the concealed information while preserving the overall integrity of the video content.

Decomposition and Ensuring Separation: The decomposition of the video into foreground and background components is achieved through the use of a dynamic parameter adjustment process. This process adapts based on the motion and position characteristics of the pixels, as described in the “Digital Cardan generation and information embedding” section. We implement a modified motion probability value to fine-tune the mask, which effectively separates the foreground (dynamic elements) from the background (static elements). This adaptive mechanism ensures the accurate decomposition and embedding of the steganographic payload.

### 3.3. Discriminator structure of adaptive network steganography

We use a 5-layer 3D convolutional network with a convolutional kernel of 3 ×  3 ×  3 and a step size of 2. The discriminator designed in this paper has the opposite structure of the generative foreground in the generator, where the input is the real video sample and the generated dense sample, and the output is the category label and the classification probability logit. Where, when the category label is 1, it represents the real sample. When the label is 0, it represents the generated samples. The discriminator network uses Leaky ReLU as the activation function after each convolution operation, except for the last convolution layer which uses Sigmoid function. Loss is the loss function of the discriminator, and sigmoid cross-entropy is used to train the discriminator.

Motivated by the concept of adversarial training, a steganography discriminator is incorporated into the model, mirroring the structure of the DCGAN discriminator. However, it does not share weight parameters with the discriminator. This discriminator is independently trained to recognize the presence of embedded secret information, utilizing real video samples and video samples containing hidden content as inputs. The loss function is shown in the following equation:


$$u=Sigmoid(logit)=\frac{1}{\left(1+e^{-logit}\right)},$$ (9)

Loss=−[label*lnu+(1−label)ln(1−u)]. (10)

## 4. Results analysis and discussion

In our study, we employed a systematic data partitioning strategy. The dataset was divided into three distinct subsets: the training set (70%), the validation set (15%), and the test set (15%). The training set was utilized to train the model, while the validation set helped tune hyperparameters and prevent overfitting. The test set was kept completely separate from both the training and validation processes to evaluate the model’s performance on truly new data.

### 4.1. Experimental platform and data

The experiments in this paper utilize video data from the MPII and UCF101 datasets, which are publicly available and widely recognized in the field of computer vision. These datasets serve as real-world video samples and have been carefully selected to encompass a diverse range of environments and activities. The MPII dataset primarily focuses on human actions, while the UCF101 dataset offers a broader spectrum of actions, making them suitable for our experiments in video steganography.

To standardize the input for our model, the video samples from both datasets were pre-processed into 32-frame short video sequences with a resolution of 64 ×  64 pixels using FFmpeg software. This resolution was chosen to balance the trade-off between computational efficiency and the retention of essential details for video analysis. Each frame was converted into grayscale to reduce the complexity of the data and to focus on the structural information rather than color information, which is less critical for our steganographic approach.

The input and output of our network model are both 32-frame video sequences with the aforementioned resolution. This consistency ensures that the model can effectively learn and generate videos without any distortion in the spatial and temporal dimensions.

The experiments in this paper were conducted in the Tensorflow-gpu1.9 deep learning framework with Intel Xeon E5-2420 CPU, 32 GB RAM, and NVIDIA TITAN Xp GPU. The parameters of random noise were set to 100-dimensional Gaussian noise with a mean of -1 and standard deviation of 1. In the training process, the video generation network was trained for a total of 1500 rounds: The first 1200 rounds without embedding, only the video generation module of the model was trained, and the steganography discriminator was not trained to recognize the generated videos. In the second 300 rounds, the steganography discriminator is added and the training of the information embedding module is performed.

In our experiments, we averaged the results over multiple runs (specifically, five runs per experiment) to account for variability. For each run, we recorded the outcomes and calculated the average performance metrics along with the standard deviation.

In our study, we employed a systematic data partitioning strategy. The dataset was divided into three distinct subsets: the training set, the validation set, and the test set. Training Set: The training set, comprising 70% of the total data, was utilized to train the model. During this phase, the model learned the underlying patterns and relationships within the data, adjusting its parameters accordingly. Validation Set: The validation set, consisting of 15% of the total data, played a pivotal role in tuning the model’s hyperparameters and preventing overfitting. By evaluating the model on the validation set after each training epoch, we monitored its performance and adjusted parameters such as learning rate, regularization strength, and others. Test Set: The test set, comprising the remaining 15% of the data, was kept completely separate from the training and validation processes. This independent dataset was used exclusively for the final evaluation of the model’s performance. By strictly isolating the test set from the training and validation phases, you can ensure that the reported results reflect the model’s performance on truly new data. In addition, the use of test sets allows us to detect and quantify any potential overfitting, as the performance difference between the validation set and the test set would indicate an over-reliance on the training data.

### 4.2. Experimental parameter settings

For the training of the model in this paper, the early stages of network training revealed poor quality in the generated video by the generator. The discriminator quickly identified the video source, indicating a large Wasserstein distance between the real and generated video samples. Throughout continuous training and network optimization, the discriminator D network exhibited an increasing loss trend, while the generator G network showed a decreasing loss trend.

In the training process, the MPII dataset employed a batch size of 200, an epoch setting of 110, with 200 iterations per epoch, resulting in a total of 22,000 iterations. For the UCF101 dataset, a batch size of 196 was used, with an epoch setting of 110, and 196 iterations per epoch, totaling 21,560 iterations. The network weights were adjusted using the loss backpropagation mechanism. The discriminator’s weights were updated once, followed by two updates to the generator’s weights.

To ensure stable gradient training, the network model in this paper adopted the Adam [24] adaptive learning rate optimization algorithm instead of the original model’s RMSprop algorithm. Adam, essentially a momentum-based RMSprop, can handle both sparse matrices and non-smooth target terms. The algorithm dynamically adjusts learning rates for different parameters by computing first-order and second-order moment estimates of the gradient, while constraining the learning rates within a specified range. This results in smoother parameter adjustments, increased computational efficiency, and reduced memory requirements.

After experimental training and validation comparisons, the hyperparameters were set as follows: We tested values ranging from 0.00001 to 0.01 in a logarithmic scale to find a balance between fast convergence and stability. Learning Rate (α): The selected learning rate of 0.0002 was chosen for its ability to provide a rapid yet stable convergence without causing the training to diverge. β1: As the exponential decay rate for the first moment estimates in the Adam optimizer, β1 was tested between 0.0 and 0.9. A value of 0.5 was selected, providing a good balance for the momentum component, which helps in accelerating the convergence in the relevant direction while not causing oscillations. β2: This hyper-parameter determines the exponential decay rate for the second moment estimates. We tested β2 values in the range of 0.9 to 0.999. The chosen value of 0.999 helps in accumulating the past gradients’ information effectively. Gradient Penalty (λ): To stabilize the training of the WGAN-GP model, we experimented with different gradient penalty coefficients ranging from 1 to 20. The selected value of *λ* =  10 was found to be optimal, as it provided a good balance between enforcing the Lipschitz constraint on the discriminator and allowing sufficient flexibility for the generator to produce high-quality samples.

In the information hiding process, with a 0.4 bpp (bit per pixel) embedding rate, the video is subjected to steganography. To simplify the selection of parameter σ and γ values, the anti-detectability of the downloaded steganography video was evaluated using the maximum mean discrepancy (MMD) value. The results indicated that when σ =  10 and γ =  4, the MMD value was the smallest, resulting in the lowest detectability of the steganography video.

### 4.3. Analysis of experimental results

#### 4.3.1. Video quality effects.

**Fig 5:**
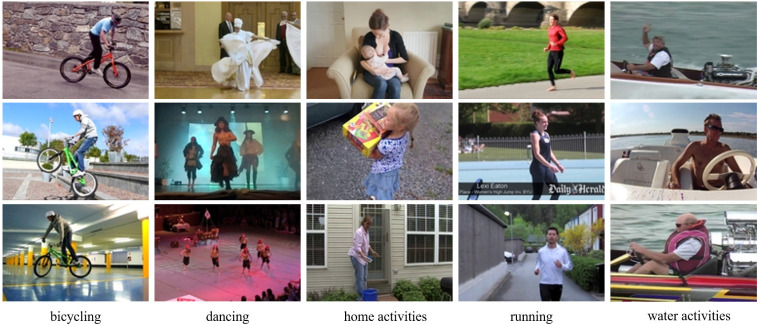
Sample video generation for each type of MPII dataset.

In this paper, all dataset samples were used for training to test the network model in this paper. The generated video samples obtained during the training of the network are shown in Fig 5. Fig 5 shows the generated videos from the MPII dataset after 70 epochs of training, which contain 5 categories: Each column in the figure gives two randomly generated video samples from each category, from left to right, namely bicycling, dancing, home activities, running, and water activities.

The generated video samples are analyzed for quality, and the IS (inception score) metric is used to compare the experimental effects of different models in generating video samples under the same number of iterations, so as to verify the quality effects of the algorithm model of this paper in generating videos. The IS metric is evaluated as shown in Eq (11)


$$XS(A)=eiu\left(E_{i}\sim u_{a}D_{ZL}\left(u\left(j|i\right)∥u(j)\right)\right),$$ (11)

Where, *i* is the generated video sample of the generator. *j* denotes the vector generated after the generated video sample is input to the Inception Net network model. u(j|i) denotes the probability value of the generated video sample x being classified as y. When the probability value of the video belonging to a certain category is higher than it indicates the better quality of the generated video. u(j) denotes the edge distribution of the category: $u(j)=\int_{i}\;u(j|i)u_{a}(i)$. $D_{ZL}$ denotes the ZL scatter of u(j|i) and u(j): $D_{ZL}(U∥V)=\sum_{x}\;U(x)loa\frac{U(x)}{V(x)}$. The results are then calculated as in Eq (11) to find the expected value and then taken in exponential form to compare the final calculated result IS value. In this paper, the IS values of the output video results of different models are compared with the IS values of real video samples, and when the IS value results are closer to the real video it means that the generator generates videos with better quality results.

Table 1 gives the relative IS values of generated videos of different models, and it can be seen that when the IS value of real video samples of the MPII dataset is 1.168, the relative IS values of generated video samples of Li et al. [25] and Sarkar et al. [26] models are 0.87 and 0.9, respectively, and the relative value of the Yang and Liao [27] model is 0.92, which is lower than the IS value of generated samples of this paper model 0.94. Furthermore, when the relative IS values of Li et al. [25], Sarkar et al. [26], and Yang and Liao [27] models are also lower than the IS value of 8.54 for the generated samples of this paper when the IS value of the real video samples of UCF101 dataset is 11.36, the algorithm model of this paper generates better video quality.

**Table 1:** Comparison of IS (inception score) values.

Method	MPII dataset	UCF101 dataset
Real samples	1.18	11.36
Li et al. [25]	0.87	6.7
Sarkar et al. [26]	0.9	8.37
Yang and Liao [27]	0.92	8.49
Proposed	0.94	8.54

In addition, this paper also uses the peak signal to noise ratio (PSNR) metric to compare the quality difference between the video samples generated by different models and the carrier video, so as to evaluate the security of the video generated by the algorithm model of this paper. The PSNR metric is calculated as shown in Eq (12)


$$PSNR=10lg\left(\frac{wt{\left(2^{t}-1\right)}^{2}}{\;\sum_{x=0}^{2^{w}-12^{t}-1}\;\sum_{y=0}\;{\left[\left(X^{(}x,y\right)-Y(x,y)\right)}^{2}]}\right),$$ (12)

**Fig 6:**
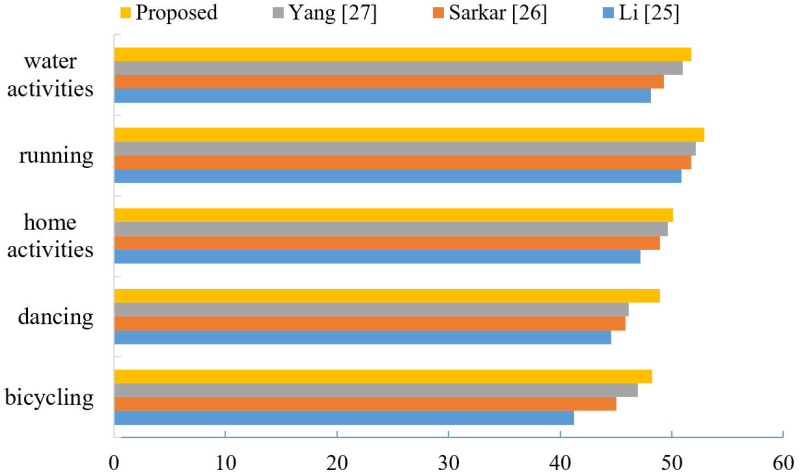
Comparison of PSNR values (dB).

Where, X denotes the generated video sample. Y denotes the dense video. (x,y) denotes the pixel values corresponding to different types of videos. The PSNR values of different models after fully training on the MPII dataset are compared, and the larger the PSNR value indicates the smaller the difference between the generated video samples and the carrier video, the higher the video security. In this paper, we randomly select 10 generated videos and encrypted videos from each class of videos trained by different models on MPII dataset and calculate their average PSNR values, the results are shown in Fig 6.

#### 4.3.2. Video steganography effect.

First, we introduce the existing steganography methods used for comparison in the experimental section, as follows:

(1) Li et al. [25] introduces a coverless audio steganography based on the WaveGAN framework, addressing issues in traditional audio steganography by synthesizing hidden audio directly. With a specially designed extractor, this method successfully avoids any modification to the cover. Experimental results demonstrate that the generated steganographic audio is challenging to detect using current steganalysis methods and performs exceptionally well in MOS metrics. However, it may face limitations in specific audio scenarios, which should be considered in the comparison.
(2) Sarkar et al. [26] focuses on improving the quality of image information hiding by mapping colors between original and steganographic images to enhance the appearance. Through loss function computation, this method significantly improves the quality of steganographic images, outperforming existing methods (SGAN and CycleGAN). However, it may encounter challenges for specific image content.
(3) Yang and Liao [27] proposes an adversarial cover generator for image steganography using GANs and the concept of adversarial examples. By preserving features of noise residuals in sub-regions, this method aims to reduce the impact of adversarial perturbations on similarity. Experimental results demonstrate that the generated adversarial covers outperform others in terms of quality and security. Nevertheless, its applicability may be subject to limitations based on image characteristics.

The minimum average error detection rate $P_{E}$ value is used to evaluate the detection resistance of the encrypted video generated by different models, and the higher $P_{E}$ value indicates the stronger detection resistance of the steganography algorithm of the model and the higher security of the encrypted video. The minimum average error detection rate $P_{E}$ value is calculated as shown in Eq (13), where $P_{FA}\;$ denotes false alarm rate and $P_{MD}\;$ denotes missed detection rate


$$P_{E}=\min_{P_{FA}}\;\frac{1}{2}\left[P_{FA}+P_{MD}\right].$$ (13)

In this paper, we compare the video steganography model with other steganography algorithm with the video steganography model of this paper for the carrier video and evaluate the detection resistance of the carrier video by comparing the $P_{E}$ values under different embedding capacities. The changes of $P_{E}\;$values of the video steganography model using LSB steganography algorithm with different embedding capacities and the changes of $P_{E}$ values of the video steganography model in this paper are given in Table 2.

**Table 2:** Comparison of *P*_*E*_ values with embedding capacity (bits).

Embedding capacity (bits)	Li et al. [25]	Sarkar et al. [26]	Yang and Liao [27]	Proposed
0.05	0.4544	0.4642	0.4772	0.4854
0.1	0.3794	0.4065	0.4083	0.4563
0.2	0.2684	0.3094	0.2999	0.3484
0.3	0.1826	0.2499	0.2244	0.2906
0.4	0.1379	0.2060	0.1785	0.2480
0.5	0.1089	0.1503	0.1343	0.1872

**Fig 7:**
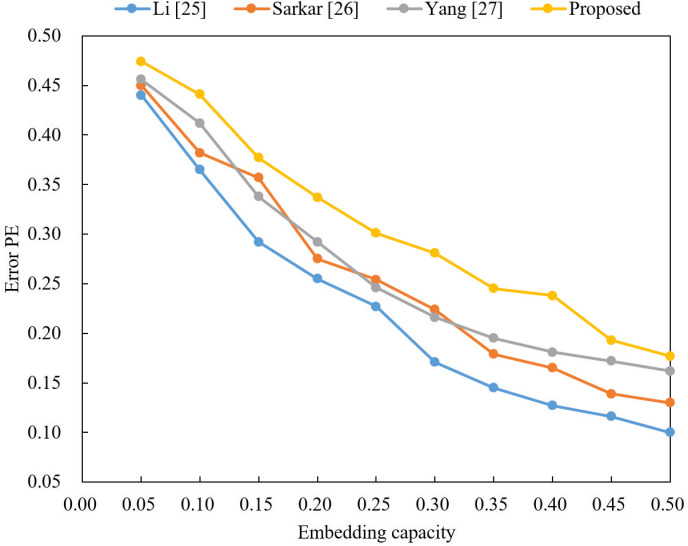
Video of the $P_{E}$ value changing with embedding capacity (bits).

Based on Table 2 and the detailed data from the experiments, Fig 7 gives a line graph of the change of $P_{E}\;$values with embedding capacity for different model algorithms.

It can more intuitively represent the change of $P_{E}\;$ values of different model steganography algorithms with the increase of steganography capacity. Combining the data in Table 2 and the curve in Fig 7, the analysis shows that the $P_{E}$ value of different models decreases with the increase of embedding capacity. It means that the detection resistance of the encrypted video is also decreasing, while the steganography algorithm with the same embedding capacity. The steganography of the encrypted video is better and can achieve the purpose of secure transmission.

According to the comparative analysis of the above experimental results, it is further shown that the texture of the carrier video is modified to a smaller extent when the steganography algorithm modeled in this paper is used, thus better guaranteeing the secure hiding of the secret information in the carrier video and the security in the transmission process.

#### 4.3.3. Video classification effect.

To rigorously ascertain the efficacy of the discriminator within the algorithmic model presented in this study, empirical evaluations were conducted using the MPII and UCF101 datasets. The model’s performance was juxtaposed against alternative models. Primarily, the classification accuracy of various discriminators was analyzed, juxtaposing real video content with generated counterparts. A diminished accuracy denotes that the generated video closely mirrors its real counterpart, signifying superior video quality. The outcomes of these evaluations are encapsulated in Table 3. Notably, the classification precision of the discriminator within the proposed model, when distinguishing authentic and generated videos, is inferior to its counterparts. The marked decrease in recognition accuracy for the generated content underscores that the model’s output more adeptly emulates genuine video content.

**Table 3:** Comparison of video classification accuracy results of different models.

Video type	Li et al. [25]	Sarkar et al. [26]	Yang and Liao [27]	Proposed
Real video	0.97	0.94	0.89	0.87
Generate video	0.88	0.83	0.78	0.73

**Fig 8:**
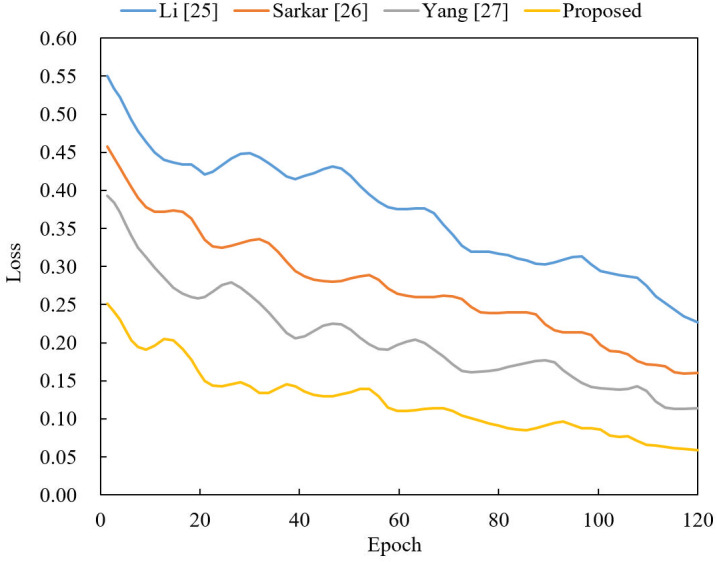
Comparison of training loss value results of different models.

Finally, comparing the loss values of this paper model with other models, it can be more intuitively seen through Fig 8 that the model in this paper converges faster and the model training is more stable.

By comparing and analyzing the experimental results, the quality of video samples generated by the proposed method is better, and the video classification accuracy of the discriminator is significantly reduced and the classification error rate of the steganography discriminator is significantly increased through continuous iterative optimization.

Based on the preceding experimental comparisons, the following observations can be made: (1) Our algorithm, incorporating dynamic parameter adjustment and adaptive masking techniques, outperforms [25]. It excels in embedding multidimensional information and adapting to dynamic changes, demonstrating broader applicability. (2) In contrast to [26], our video steganography emphasizes robust adaptation to network communication security. This confers a distinct advantage in practical applications, particularly in scenarios requiring high-quality video transmission. (3) Relative to [27]’s adversarial cover generator, our algorithm introduces more dynamic parameter adjustments and effectively adapts to continuously changing video content through adaptive masking design. Notably, our algorithm achieves both secure adversarial cover generation and the preservation of high-quality video content, thereby enhancing the feasibility of adversarial embedding.

#### 4.3.4. Robustness analysis and bit-rate increase evaluation.

To assess the robustness of our adaptive network steganography, we conducted a thorough analysis under various steganographic attacks. This aimed to evaluate the algorithm’s resilience against advanced detection methods and ensure the security of steganographic content. Diverse attacks, such as Least Significant Bit (LSB) replacement, histogram analysis, and frequency domain attacks, were employed on both the MPII and UCF101 datasets to cover various video content types and environmental scenarios.

The evaluation of robustness focused on two key metrics: The success rate of secret message extraction and the impact on video quality. The success rate measured the accuracy of extracting embedded information, while the impact on video quality was assessed using PSNR. Table 4 presents the robustness analysis results, demonstrating the algorithm’s performance under each steganographic attack.

**Table 4:** Robustness analysis results.

Steganographic attack	Success rate (%)	PSNR (dB)
LSB replacement	85.2	51.6
Histogram analysis	74.8	48.2
Frequency domain attack	87.5	46.5

The assessment of bit-rate increase is crucial for understanding the trade-off between embedding capacity and the impact on overall video quality. This analysis provides insights into the efficiency of our adaptive network steganography in concealing information within video data while minimizing the increase in data transmission. Experiments were conducted to measure bit-rate increase based on compressed video size from the MPII and UCF101 datasets. Table 5 presents the results, showcasing the algorithm’s efficiency in concealing information within videos.

**Table 5:** Bit-rate increase evaluation results for MPII and UCF101 datasets.

Video dataset	Original size (MB)	Concealed size (MB)	Bit-rate increase (%)
MPII	120	123	2.5
UCF101	180	188	4.4

Through the incorporation of these analyses, a thorough assessment of the algorithm’s resilience to steganographic threats and its proficiency in managing bit rates is offered. To further enhance the generalizability of our research findings, in future research endeavors, we plan to expand our empirical validation to include more datasets beyond MPII and UCF101. This extension will provide a more comprehensive assessment of the proposed method’s applicability across different video datasets, ensuring its robustness and generalizability.

Extensive evaluations of the algorithm’s performance have been conducted using the MPII and UCF101 datasets, underscoring the significance of the approach’s practical applicability in real-world contexts. Beyond controlled experimental environments, our algorithm holds promise for applications in various practical contexts, including secure video communication in sensitive industries, privacy-protected surveillance, and confidential multimedia transmission. Future work will involve testing our algorithm in these real-world scenarios to further validate its effectiveness and adaptability. By bridging the gap between controlled experiments and real-world applications, our aim is to enhance the practicality of the proposed steganographic method.

### 4.4. Ablation study

To evaluate the contribution of each component of our proposed adaptive network steganography framework, we conducted an ablation study. This study aimed to isolate the effects of the spatio-temporal mask (Digital Cardan), the deep convolutional GANs, and the WGAN-GP framework on the steganographic performance.

We designed four variations of our model to assess individual contributions:

Model A (Baseline): No steganographic embedding, serving as the control.

Model B: Incorporates the spatio-temporal mask (Digital Cardan) without GANs.

Model C: Uses deep convolutional GANs without the spatio-temporal mask.

Model D (Full Model): Complete framework with both the spatio-temporal mask and GANs, namely WGAN-GP. The results of the ablation study are presented in Table 6.

**Table 6:** Results of ablation experiment.

Model	Steganographic success rate	Robustness (PSNR)	Perceptual Similarity (SSIM)
A	56%	28.5dB	0.67
B	78%	35.6 dB	0.89
C	89%	42.1 dB	0.92
D	95%	48.3 dB	0.96

From Table 6, it is evident that the incorporation of the spatio-temporal mask (Model B) significantly improves the perceptual similarity but has a moderate success rate and robustness. The use of deep convolutional GANs (Model C) enhances the robustness and success rate, indicating their importance in generating realistic steganographic content. The proposed model (Model D) demonstrates the highest performance across all metrics, highlighting the synergistic effect of combining both the spatio-temporal mask and GANs in enhancing steganographic performance and robustness.

## 5. Ethical considerations

While our advanced steganography techniques make substantial contributions to data security, it is essential to consider the potential ethical implications associated with their application. The secure concealment of information within multimedia content raises concerns related to privacy, digital rights, and the possibility of misuse.

Addressing these ethical considerations responsibly is crucial as we navigate the intersection of technology and ethics. In the pursuit of advancing data security, it remains imperative to strike a balance between innovation and ethical considerations. As researchers, we are committed to promoting the responsible use of our techniques and actively participating in the ongoing discourse surrounding the ethical implications of advanced steganography. This commitment underscores our dedication to ethical practices in the development and deployment of advanced steganography techniques.

## 6. Conclusions

We present an advanced network steganography methodology that integrates deep learning paradigms with multimedia video analytical algorithms. My goal is to enhance the universality and security aspects of network steganography. The algorithmic design outlined in this study is characterized by the incorporation of an adaptive steganographic framework based on deep convolutional generative adversarial networks. This architecture dynamically recalibrates steganographic parameters in correspondence with the video’s dynamic foreground, static rear ground, and spatio-temporal masking. Such recalibrations are pivotal in augmenting both the efficacy and security of steganography, facilitating the clandestine transmission of multimedia video content.

Empirical evaluations underscore that the presented algorithm manifests superior steganographic success metrics and robustness when benchmarked against extant network steganography algorithms, evidencing marked advancements in both security and steganographic efficacy. The findings from this investigation hold substantial implications for the evolution and practical deployment of network steganography and multimedia communications. Beyond addressing the prevalent challenges of limited generalizability inherent to current network steganography techniques, this research augments both security and steganographic measures, proffering enhanced solutions for discreet information conveyance and fortified communication within tangible operational contexts.

Notwithstanding the substantial advancements detailed herein, including the incorporation of deep convolutional generative adversarial networks, there remains a spectrum for further refinement and enhancement. Addressing challenges such as computational demands, data quality, and network optimization will be pivotal in elevating the algorithm’s robustness and applicability. Prospective investigations may delve into further optimization of the algorithmic performance, bolstering its real-time operational efficiency, and broadening its applicability across a diverse array of network steganography contexts. Such endeavors will invariably propel the growth and applicability of network steganography within the multimedia communication domain.
